# Joining Chemical Pressure and Epitaxial Strain to Yield Y-doped BiFeO_3_ Thin Films with High Dielectric Response

**DOI:** 10.1038/srep25535

**Published:** 2016-05-09

**Authors:** N. D. Scarisoreanu, F. Craciun, R. Birjega, V. Ion, V. S. Teodorescu, C. Ghica, R. Negrea, M. Dinescu

**Affiliations:** 1 National Institute for Laser, Plasma and Radiation Physics, 077125 Magurele, Romania; 2 CNR-ISC, Istituto dei Sistemi Complessi, Area della Ricerca di Roma-Tor Vergata, Via del Fosso del Cavaliere 100, I-00133 Rome, Italy; 3 Faculty of Physics, University of Bucharest, 077125 Magurele, Romania; 4 National Institute of Material Physics, 077125, Magurele, Romania

## Abstract

BiFeO_3_ is one of the most promising multiferroic materials but undergoes two major drawbacks: low dielectric susceptibility and high dielectric loss. Here we report high in-plane dielectric permittivity (ε’ ∼2500) and low dielectric loss (tan δ < 0.01) obtained on Bi_0.95_Y_0.05_FeO_3_ films epitaxially grown on SrTiO_3_ (001) by pulsed laser deposition. High resolution transmission electron microscopy and geometric phase analysis evidenced nanostripe domains with alternating compressive/tensile strain and slight lattice rotations. Nanoscale mixed phase/domain ensembles are commonly found in different complex materials with giant dielectric/electromechanical (ferroelectric/ relaxors) or magnetoresistance (manganites) response. Our work brings insight into the joined role of chemical pressure and epitaxial strain on the appearance of nanoscale stripe structure which creates conditions for easy reorientation and high dielectric response, and could be of more general relevance for the field of materials science where engineered materials with huge response to external stimuli are a highly priced target.

Multiferroics (MF) are materials which have simultaneously more ferroic properties, such as ferroelectric, antiferrodistortive or magnetic order^1^. The multitude of their properties is given by the coupling between the various ferroic orders, which can boost the developing of new multifunctional devices.

Among MF, BiFeO_3_ (BFO) is certainly the most investigated due to both polar and magnetic orders coexisting at room temperature, which are of interest for potential applications in nonvolatile memories, spintronics, piezoelectric devices etc^2^. The interplay between its ferroic order parameter and the various structural degrees of freedom gives rise to a complex phase diagram^2^. Thus, below the critical temperature *T*_c_ ≈ 1100 K, BFO transforms from the paraelectric orthorhombic (*O*) Pnma phase into the rhombohedral (R) *R*3*c* ferroelectric (FE) phase. The rhombohedral *R*3*c* ground state is characterized by antiphase octahedral tilts about the [111]_c_ directions and ionic displacements from the centrosymmetric positions along the same axis, with pseudocubic lattice parameters *a* = 3.965 Å and α = 89.4^o^^3^. Below the Néel temperature *T*_N _∼ 640 K BFO has also a G-type antiferromagnetic (AFM) ordering of Fe^3+^ ions^4^.

Among the drawbacks of BFO, the small dielectric constant, high dielectric loss and high leakage currents, which are detrimental for its applications in electromechanical devices, must be pointed out. The intrinsic dielectric constant at radio frequencies was measured to be about 30^5^. At lower frequencies (kHz and MHz range) higher values (up to a few hundreds^2^) have been measured, however they decrease with frequency and are accompanied by high dielectric losses, which are the fingerprint of space charge contributions^2^. The small intrinsic dielectric value has been attributed to the far away critical temperature *T*_c_^2^.

There are two main ways to control the phase stability and transition temperatures in BFO: one is through strain engineering and the other is through chemical substitutions. Both aim to induce, by different means (mechanical pressure or chemical pressure) a mixed state, characterized by coexisting phases, in order to improve its dielectric and electromechanical response. The two degrees of freedom, polar displacements and oxygen octahedral tilts which coexist in a wide temperature range, can react differently to chemical substitutions or external parameter variations and thus lead to a multitude of phases. Thus BFO is very sensitive to the application of an external pressure: above 10 GPa the pressure brings the high temperature phase (*O* Pnma) at room temperature while, at lower pressures, several low symmetry (monoclinic) phases are stabilized at room temperature^6^. This is important, taking into account that the stress established within the plane of growth in epitaxial films can reach several GPa^7^. The strain can be exerted in a controlled manner in thin films and the strain engineering is currently used to tune their functional properties^8^. In thin film form the crystallographic structure of epitaxial thin films often deviates from the bulk due to strain. For instance BFO films grown on low misfit substrates adopt a monoclinic (*M)* structure for compressive strain^9^,^10^. Thus BFO epitaxial films with thicknesses in the range 40–960 nm grown on SrTiO_3_ (STO) (001) substrates^11^ change from angularly distorted tetragonal-like to monoclinic and finally to more bulk-like distorted rhombohedral structures as the strain relaxes with increasing thickness. Similar results have been reported by Saito *et al*.^12^ for BFO films grown on STO (001) substrates with thicknesses varying in the range 15–500 nm. Thus for films with thickness below 50 nm a fully strained structure with tetragonal symmetry was obtained, while for more relaxed films (with thickness above 50 nm) a monoclinic structure with continuously varying lattice parameters was observed. Remarkably, a rhombohedral bulk-like structure was not found even for the thickest films. Similar results have been reported by Kan and Takeuchi^13^. BFO films with thicknesses 50 nm–1000 nm grown on STO (001) showed monoclinic symmetry with different lattice parameters, depending on film thickness, thus on the degree of strain relaxation.

As previously stated, an alternative way to tune materials at the boundary between different but energetically equivalent structural phases is through chemical substitutions. As a consequence, huge response at weak external signals can be obtained in the presence of an energy landscape of multiple phases close in energy^14^.

Recent research on doped BFO focused on structural and electromechanical properties of rare earth (RE) substituted BFO thin films^15^,^16^. A strong dependence on the ionic radius of the substituted elements has been observed. When rationalized in terms of the average A-site radius, a R-O structural transition was observed at the same average radius value, independently of the RE ion type. Based on results obtained on doping with different concentrations of RE ions, a universal phase diagram in terms of ionic radius has been proposed^17^,^18^.

The size of RE ions controls also the stability of the structural phase, as shown by the temperature dependence of the lattice parameters^17^. Thus, a stronger chemical pressure, induced by the smaller ionic radius of the substituting RE elements extends the orthorhombic phase towards lower composition and temperature range. Transmission electron microscopy (TEM) on RE-BFO substituted films evidenced the presence of different competing phases in a narrow composition range associated with a morphotropic phase boundary (MPB). The MPB is related to a structural transition from the FE *R* phase to an *O* phase, with enhancement in the dielectric constant and piezoelectric coefficient. For example in Bi_1−x_Sm_x_FeO_3_ films grown on STO (001) substrates the MPB around room temperature was found for compositions with x ≅ 0.14^16^. For smaller radius RE dopants (RE **=** Gd^3+^, Dy^3+^) the MPB occurred at smaller x values (0.12 for Gd and 0.07 for Dy). In all Bi_1−x_RE_x_FeO_3_ compositions it was found that the transformation from the *R* phase to the O phase at MPB is associated with a nanoscale phase mixture composed of the parent BFO *R* lattice and lamellar ferroelectric nanodomains with alternating polarizations^16^,^19^. Thus the occurrence of such complex phase coexistence was considered responsible for the enhanced dielectric and piezoelectric response at MPB, by providing a low energy pathway for the polarization transition between the different polar axes. Indeed an enhanced dielectric constant (∼300) was measured for MPB Bi_1−x_RE_x_FeO_3_ (RE **=** Sm, Gd, Dy) compositions at room temperature^17^. These results have been obtained on films with 200 nm thickness entirely relaxed, thus the epitaxial strain was playing no role in their properties^17^.

We take further this investigation and address the problem of concomitant influence of RE-doping and epitaxial strain on BFO properties. For doping, a RE ion (Y^3+^) with smaller ionic radius has been chosen. We show that Y-BFO films with thickness about 100 nm display very high in-plane dielectric constant. We relate this huge enhancement to the destabilizing influence of the chemical pressure induced by the small size ion substitution and the peculiar nanostripe formation due to partial strain relaxation. Our results can be of more general relevance for other materials where properties improvement is pursued through the combined role of chemical pressure (doping) and epitaxial strain.

## Results and Discussion

We have employed pulsed laser deposition (PLD) technique to grow Bi_1−x_Y_x_FeO_3_ (Y-BFO) epitaxial thin films with thickness of about 100 nm on STO (001) single crystal substrates. For comparison, pure BiFeO_3_ (BFO) thin films have been grown also, on the same type of substrates. As further described in the Experimental section, a relatively low growth rate (∼0.1 Å/s) and high deposition temperature (∼700 ^o^C) have been selected for deposition. The deposition conditions have a strong influence on the final lattice parameters and tetragonality of the BFO films. Indeed it has been shown that tetragonal-like BFO films can be grown even on low-mismatch substrates such as STO by controlling the growth rate and deposition temperature^20^,^21^,^22^,^23^. Namely, at low growth rates and/or high substrate temperature the incident adatoms can diffuse more easily and follow the substrate lattice, while at high growth rates or low deposition temperature the arriving adatoms will accumulate in island-type zones where a high strain state is created. These high strain areas favour the tetragonal symmetry of the films, with high tetragonality ratio^21^. However, we have preferred to work with a low growth rate and high deposition temperature in order to promote a layer-by-layer-like growth and smooth surface.

**Figure 1:**
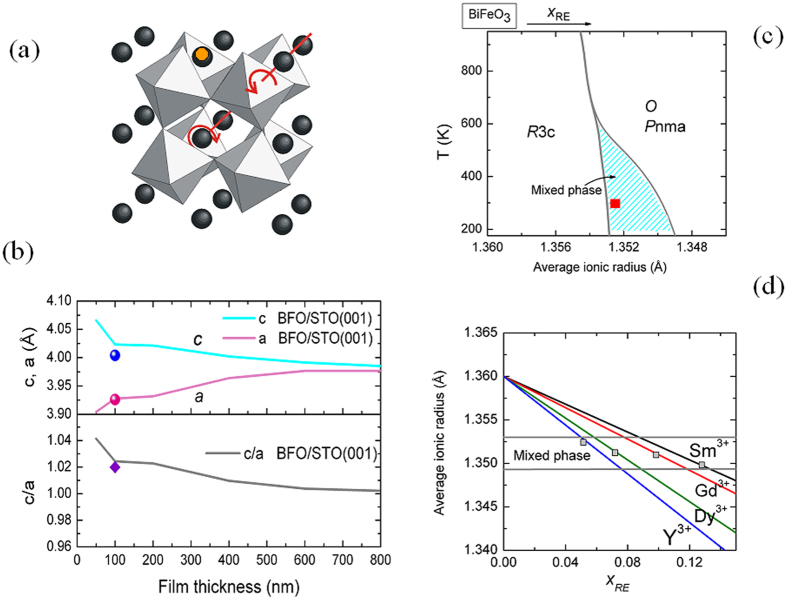
(**a**) Schematic view of the tilt distortions in the ground R3c structure of a RE-doped BFO, where the black spheres in the interstices between oxygen octahedra represent Bi^3+^ ions, while the smaller (orange) sphere is the RE ion; (**b**) Lattice constants and tetragonality ratio of Y-BFO films, represented by points. For comparison, the curves of variation of the BFO parameters with film thickness, as reported in ref. 13, are also shown; (**c**) A schematic of the universal phase diagram of RE-doped BFO, as reported in refs 16,17. The red square represents the selected composition for our films, in terms of average ionic radius; (**d**) Average ionic radius - *x* dependence, for different RE elements, calculated as described in the text.

The selected composition was Bi_0.95_Y_0.05_FeO_3_. Figure 1(a) shows a schematic view of the tilt distortions (a^-^a^-^a^-^ system in Glazer’s notation^24^) in the ground R3c structure of a RE-doped BFO, where the black spheres in the interstices between oxygen octahedra represent Bi ions, while the smaller (orange) sphere is the RE ion.

It is clear that the smaller its radius is the larger is the available space for the tilting of oxygen octahedra in the structure and easier is the appearance of a new structural phase. Figure 1(b) shows the lattice constants of our Y-BFO films, placed in the context of a typical variation of BFO lattice constants with film thickness, as reported in ref. 13. Figure 1(c,d) shows our selected composition Bi_0.95_Y_0.05_FeO_3_ in the context of temperature - average ionic radius^17^ and average ionic radius - RE concentration diagrams. There will be more comments on these figures in the further discussion. In order to compare the properties of Y-BFO films with those of the undoped BFO films, another set of samples deposited on STO in the same conditions starting from pure BFO targets has been examined.

**Figure 2:**
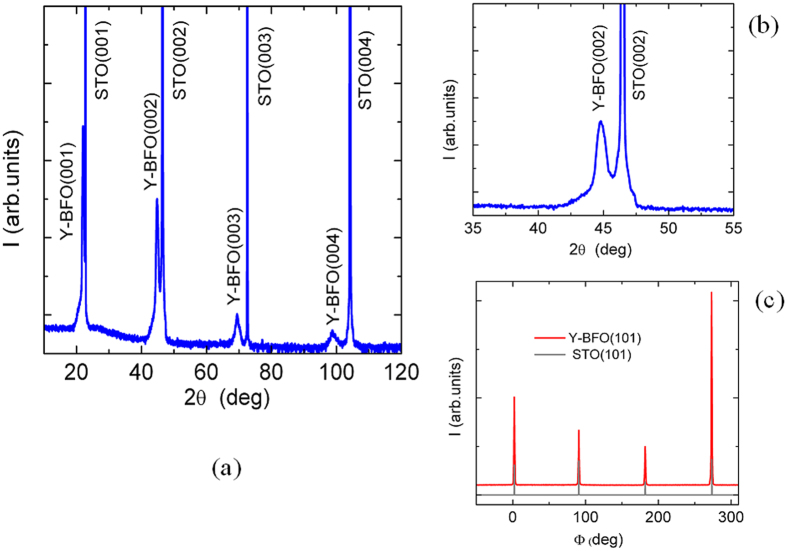
(**a**) The θ-2θ diffraction patterns of the Y-BFO films; (**b**) A detail around the (002) reflection; (**c**) XRD Φ –scans showing the (101) reflections of the Y-BFO film and STO substrate.

**Figure 3:**
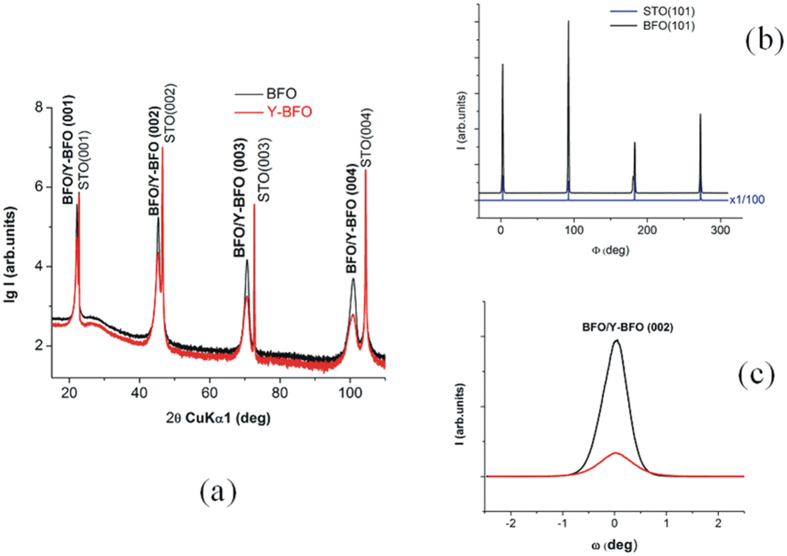
(**a**) Comparison between θ-2θ diffraction patterns of the Y-BFO and BFO films; (**b**) Comparison between Φ-scans for the same samples; (**c**) Full width at half maximum (FWHM) values of the rocking curve of the (002) diffraction peaks for Y-BFO and BFO films.

Figures 2(a) and 3(a) show the θ-2θ X-ray diffraction patterns of the Y-BFO (film 1) and BFO films. A detail around the (002) reflection is shown in Fig. 2(b). Only (00l) reflections of Y-BFO and BFO films and STO substrate are observed, demonstrating the pure phase and epitaxial growth of the films. In-plane and out-of-plane lattice parameters were determined from single scans through the symmetric (00l) and asymmetric (hkl) reflections, respectively. The structural parameters of the films (in pseudocubic notation) are listed in Table 1. The out-of-plane lattice parameter *c* has been found to be 4.0042 Å for Y-BFO film, slightly larger in comparison with the BFO film (3.9983 Å). We recall that for Bi_0.95_Y_0.05_FeO_3_ ceramics the pseudocubic lattice parameter is about 3.94 Å^25^. Thus the out-of-plane lattice parameter *c* of the Y-BFO films exceeds the bulk pseudocubic lattice parameter, while the in-plane lattice parameter *a* is about 3.9266 Å, fairly close to the cubic STO substrate lattice parameter (3.905 Å). The tetragonality ratio *c/a* has been found to be about 1.02, slightly larger in comparison with the value obtained for the BFO film. The difference between the out-of-plane and in-plane lattice constants is attributed to compressive coherent in-plane strain induced by the STO substrate which causes a cell elongation in the out-of-plane direction. In Fig. 1(b) we have compared this pseudo-tetragonal distortion with results previously reported in literature for BFO films grown by pulsed laser deposition^13^. For a film thickness of about 100 nm the lattice distortion is similar and the films are in a partially relaxed strain state. From our results we cannot say more about the cell symmetry, but there is by now a general consensus that BFO films grown on STO (001) substrates show monoclinic symmetry for a wide range of film thicknesses^13^,^26^,^27^,^28^.

The degree of in-plane orientation was assessed by the XRD Φ –scans. As shown in Figs 2(c) and 3(b) the peaks of the (101) reflection of the Y-BFO and BFO films occur at the same azimuthal Φ angles as those for STO (101) substrate reflection and are 90^o^ apart from each other. This indicates the presence of fourfold symmetry with a “cube-on-cube” epitaxial growth on the STO (001) substrate.

The full width at half maximum (FWHM) values of the rocking curve of the (002) Y-BFO diffraction peak is larger in comparison with the (002) diffraction peak of the pure BFO film due to both the monoclinic distortion in the out-of-plane direction and dopant-induced lattice modifications (Fig. 3c). Similar analysis performed on a second set of Y-BFO films (film 2) grown in the same conditions yield comparable results (see the Supplementary Information). The Supplementary Information includes XRD patterns of the high angle peak (004) of the Y-BFO (film 2) in comparison with the Y-BFO (film 1) and the reference BFO film, as well as the superimposed rocking curves of the (002) peaks.

The substitution with Y on the B-site apparently causes a decrease of the in-plane lattice parameter and an increase of c/a ratio with respect to BFO film lattice (Table 1). In order to better understand the effect of the Y-dopant on the films, an Williamson-Hall approach was used to separate the various possible contributions to the broadening of XRD reflections^29^. The method is based on the assumption that an epitaxial film consists of single crystallites, defect-free domains, named mosaic blocks, with a mean vertical and lateral dimension called vertical and lateral coherence length^29^,^30^. The terms lateral and vertical are associated to directions parallel and perpendicular to the substrate surface, respectively. The vertical coherence length is to be connected to films thickness for systems with defects running mainly parallel to the substrate surface normal. The mosaic blocks are assumed to be slightly misoriented with respect to each other, the out-of-plane rotation of the blocks being called mean mosaic tilt angle^29^,^30^. The vertical coherence length, *L*_⊥_ and the heterogeneous microstrain Bragg reflection *ε*_⊥_ are derived from the Williamson-Hall plots^29^ using the radial scan (*ω*-2*θ* scan) directions of (*00l*) symmetric reflections, by using the following relation


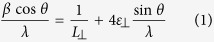


where λ is the X-ray wavelength, β - the integral breadth (instrumental corrected FWHM) and θ - the Bragg angle. The plot of (*β* cos *θ*)/*λ* versus (sin *θ*)/*λ* gives the value of the strain from the slope and the vertical coherence length from the ordinate intercept.

From the broadening of the rocking curve (*ω* scan) of the same symmetric (00*l*) reflections, using a similar Williamson−Hall approach proposed by Mentzger *et al*.^31^, the coherence length parallel to the substrate surface *L*_||_ and the tilt angle *α*_tilt_ could be extracted. All the XRD results are summarized in Table 1, revealing the effect of Y-doping. The Y-BFO films exhibit the following features in comparison with the undoped BFO film: higher microstrain values, slightly larger tilt angles and an important decrease of their lateral coherence lengths. This last aspect is to be related with the formation of smaller nanodomains parallel to the substrate.

**Figure 4:**
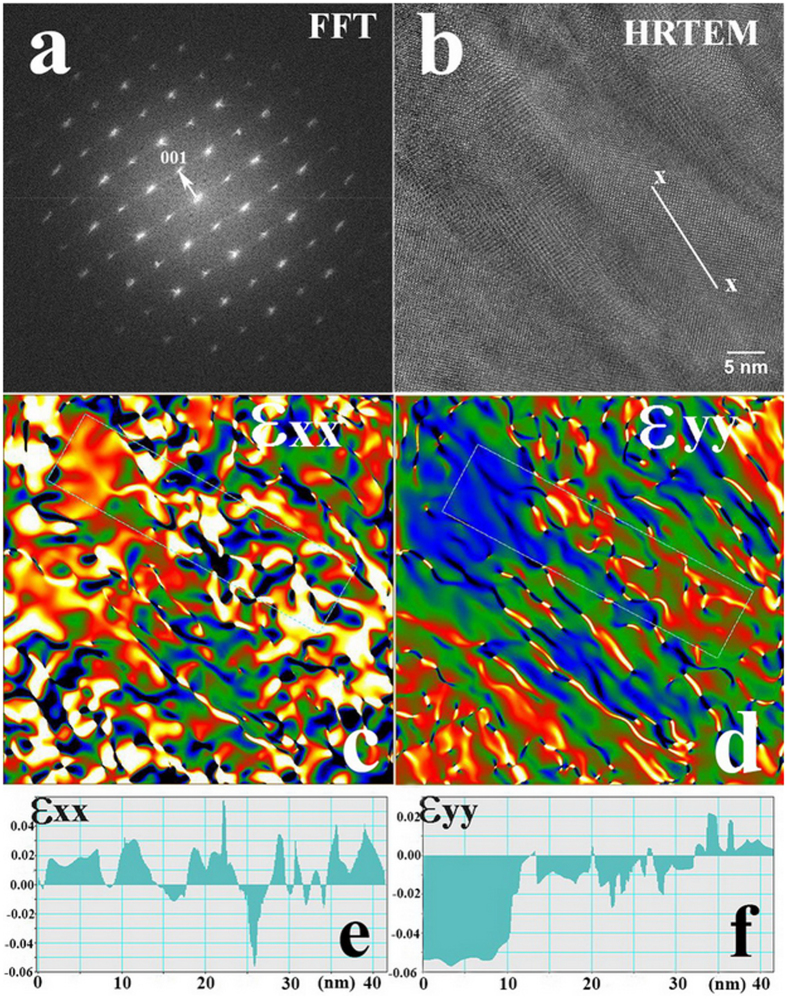
(**a**) Fast Fourier transform (FFT) pattern used for the strain analysis corresponding to the cross-sectional HRTEM image of the middle zone of the Y-BFO film shown in (**b**). (**c**) Microstrain analysis performed on the HRTEM image by geometric phase analysis (GPA) along the Ox direction; (**d**) the same for the Oy direction; (**e**), (**f**) Line profiles of ε_xx_ and ε_yy_ strains, respectively, obtained on the marked zones displayed in the previous images.

Figure 4 shows a cross-sectional HRTEM image of the Y-BFO film and the corresponding fast Fourier transform (FFT) pattern (Fig. 4a) used for the strain analysis. The HRTEM image evidences contrast variation across nanodomain stripes with widths of 5**–**20 nm, oriented approximately along the x-x direction (Fig. 4b), which has been selected along the 001 direction of the Y-BFO structure (parallel to the substrate normal). The FFT pattern of this region shows the fundamental perovskite reflections with spot splitting or diffused elongation due to the tilted stripe nanodomain structure.

A microstrain analysis has been performed on the HRTEM image by geometric phase analysis (GPA)^32^. The variation of strain has been analysed along two directions Ox and Oy oriented perpendicular (ε_xx_ in Fig. 4c) and parallel (ε_yy_ in Fig. 4d) to the substrate normal. Line profiles of ε_xx_ and ε_yy_ strains obtained on the marked zones are displayed in Fig. 4(e,f), respectively. In the strain maps the blue zones are under strong compressive strain, the red ones are under strong tensile strain while the green ones are not strained. Small ε_xx_ strain variations are observed along the stripes, alternating sign with an average “period” of a few nm. However the most interesting strain variation is found on the perpendicular direction, where an alternation of compressive and tensile ε_yy_ strains is registered across the stripes. A detailed analysis in different zones evidences small lattice rotations between different stripes. This shows that both lattice cell as well as stripe orientation variations, compatible with monoclinic distortion, occur in strained Y-BFO films.

**Figure 5:**
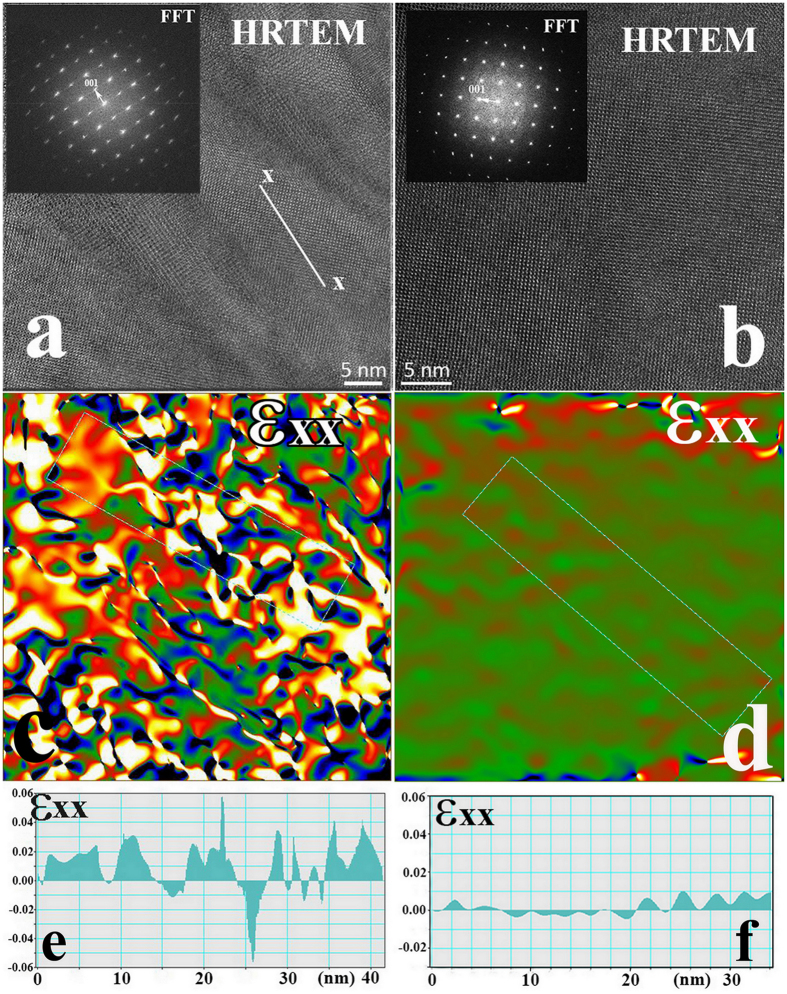
Comparative HRTEM, FFT and stress/strain GPA maps and line profiles of the Y-BFO (**a**,**c**,**e**) and BFO (**b**,**d**,**f**) films far from the interface.

Comparative HRTEM and stress/strain GPA maps of the Y-BFO and BFO films are shown in Fig. 5 (a–d). These analysis evidenced that the pure BFO films do not show stripe modulation, as confirmed by the uniform aspect of the HRTEM image (Fig. 5b), but they are still strained due to the substrate influence. The major difference is that the strain level in BFO film is lower and almost uniformly distributed (Fig. 5d). The line profile shown in Fig. 5(f) evidences that the strain variations are much smaller than in Y-BFO film (Fig. 5e), therefore we can presume that nanostripe formation occurs only for Y-BFO films. These assertions are supported also by the structural data extracted from XRD analysis (Table 1), which show that BFO thin films are characterized by lower microstrain and larger lateral coherence length values than Y-BFO films.

There are multiple evidences that, in partially relaxed films, the in-plane strain does not relax to zero but locally oscillates, by different mechanisms, including monoclinic twinning rotation^33^. Moreover, for the case when the epitaxial constraint is partially relaxed, it has been observed that the result is a nanoscale mixed-phase structure^34^, with the lattice mismatch accommodated by the gradual deformation of the structure between different phases. This nanoscale mixed-phase ensemble has been compared to the relaxor ferroelectrics and to the colossal magnetoresistance (CMR) manganites, which show similar features, and where a relatively low signal can activate a large response^34^.

**Figure 6:**
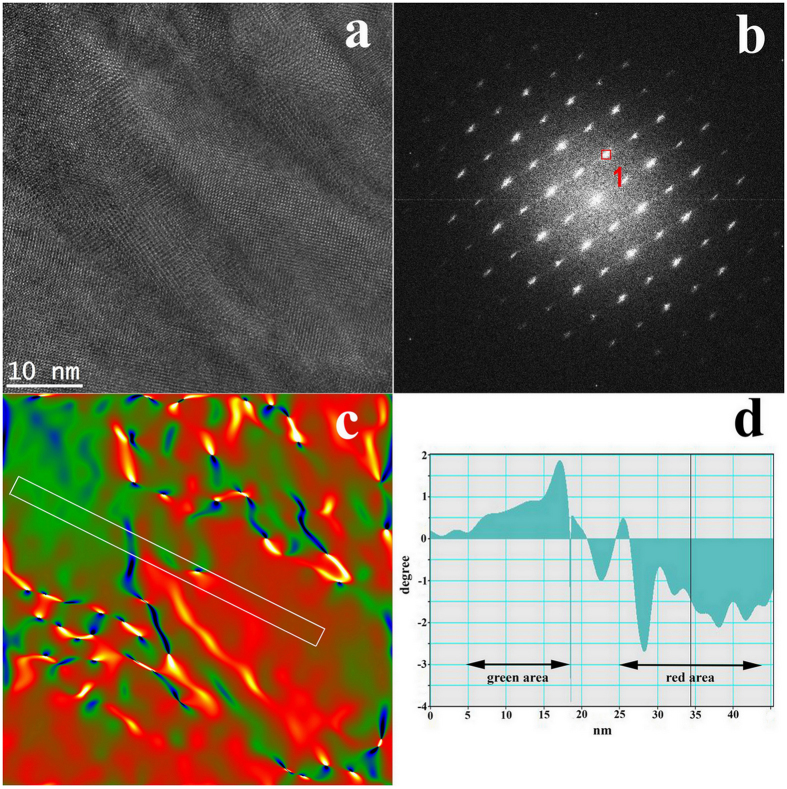
(**a**) HRTEM image of the Y-BFO film, taken on the middle region of the film; (**b**) Power spectrum corresponding to this image area; (**c**) GPA analysis performed on the same area in order to evidence local lattice rotations; (**d**) Resulting local lattice rotation along the rectangular area indicated in the phase image.

The additional distortions associated with the presence of nanodomains are related to the elastic bending and rotation of the lattice planes in order to fit the adjacent domains. They result into nanostripes with contrast difference as seen in Fig. 6(a). These nanostripes have widths of about 10 nm and an additional detailed GPA analysis has been performed in order to evidence their rotation. Thus Fig. 6 displays lattice rotation analysis on the Y-BFO film, operated on a representative HRTEM image taken on the middle region of the film, shown in Fig. 6(a), together with the power spectrum corresponding to this image area (Fig. 6b) and the phase image corresponding to the same area, calculated for the (110) reflexion in the power spectrum (Fig. 6c). The local lattice rotation was calculated using GPA^32^. The resulting lattice rotation along the rectangular area indicated in the phase image, connecting the green and the red areas, is displayed in Fig. 6(d). It can be observed that the Y-BFO lattice in the green area is rotated by about 1.5 degrees in the positive sense while the red area is rotated by about 2 degrees in the negative sense. The total rotation between the green and the red domains is about 3.5 degrees.

**Figure 7:**
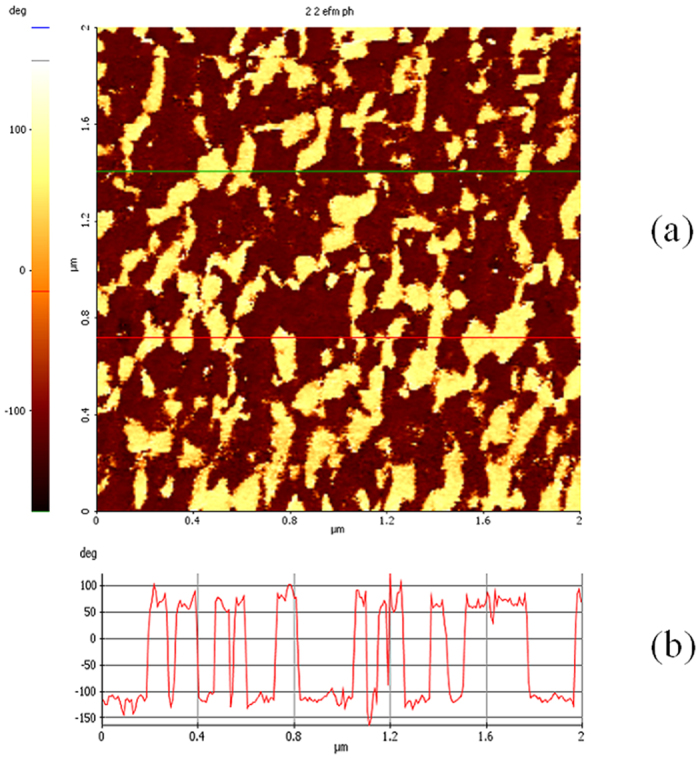
(**a**) Out-of-plane PFM phase image measured on a Y-BFO film surface. The domains are polarized into the specimen surface or out of the sample, as indicated by the dark or light normal PFM phase contrast, respectively; (**b**) The line profile shows that the phase difference is nearly 180^0^.

In Fig. 7(a) a piezoforce microscopy (PFM) phase image measured on a Y-BFO film surface is shown, together with a typical line profile (Fig. 7b). The out-of-plane PFM response shows strong domain contrast, indicating that the material is polar with a polarization vector oriented mainly along the c-axis. Domains are identified as being polarized either into the specimen surface or out of the sample, depending on dark or light normal PFM phase contrast, respectively. The line profile shows that the phase difference between the zones with different contrast is nearly 180^°^.

**Figure 8:**
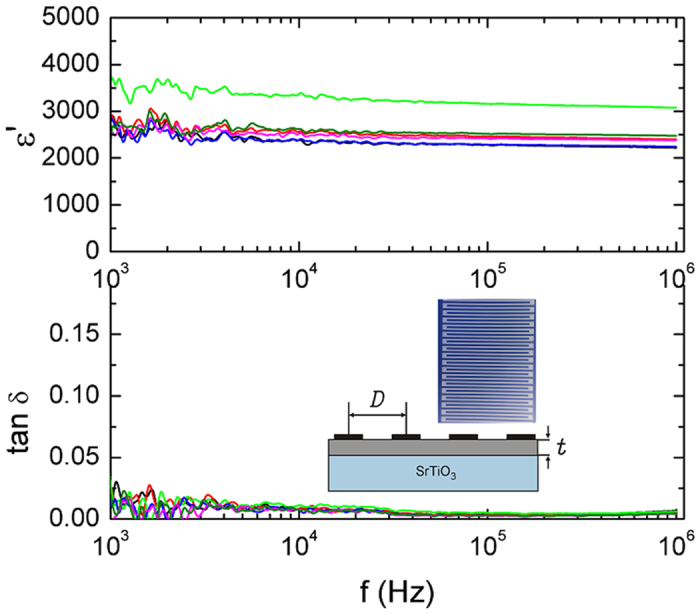
Y-BFO dielectric constant and loss tangent values, measured on 6 representative IDE in the frequency range 1 kHz-1 MHz. A sketch of the IDE structure is shown in the inset.

Dielectric spectroscopy measurements have been made on interdigital electrodes (IDE) deposited on top surfaces of the films (10 IDE structures on each sample). A sketch of the IDE structure is shown in the inset in Fig. 8. The measurements yield the capacitance and the dielectric loss tan δ. Each IDE consists in N **=** 21 finger pairs of length L **=** 464 μm and width 10 μm. The interspace between fingers is 10 μm, thus the distance D between finger centres is 20 μm. A second set of similar samples but provided with IDE of different dimensions (N **=** 15, L **=** 1200 μm, width **=** 7. 5 μm, D **=** 15 μm) has been prepared and investigated, in order to rule out the possible influence of IDE characteristics on the measured values.

The in-plane dielectric constant of the Y-BFO thin film capacitors with the interdigital electrode configuration was calculated by using the analytical model derived by Farnell *et al*.^35^ and further developed in ref. 36. It has been shown that the dielectric constant of the thin film ε can be calculated using the Eq. (2)


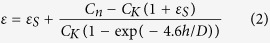


where ε_S_ is the dielectric constant of the substrate, h is the film thickness (h **=** 100 nm), C_K_ is a constant depending on IDE geometry and C_n_ is the measured capacitance of the IDE normalized to the finger length (L) and to the number of fingers (2N−1). In the case of IDE patterns with equal finger width and spacing the constant C_K_ **=** 4.53 pF/m^35^,^36^. The dielectric constant of the STO substrate is ∼300.

The Y-BFO dielectric constant and loss values measured on 6 representatives IDE are plotted in Fig. 8. The values measured at 10 kHz are about 2500 (on one transducer a value >3000 was obtained). The dielectric loss is very small (∼0.007). Moreover, neither the losses nor the dielectric constant show significant variation in the frequency range 1 kHz–1 MHz.

The values obtained for the dielectric constant are by far the highest obtained for BFO, bulk or thin film, pure or doped. In order to verify the experimental procedure and the calculation method, measurements have been repeated on IDE electrodes directly deposited on the substrate and the same model has been applied to calculate the dielectric constant. Perfect agreement has been obtained between the measured and the data sheet value of the STO dielectric constant.

**Figure 9:**
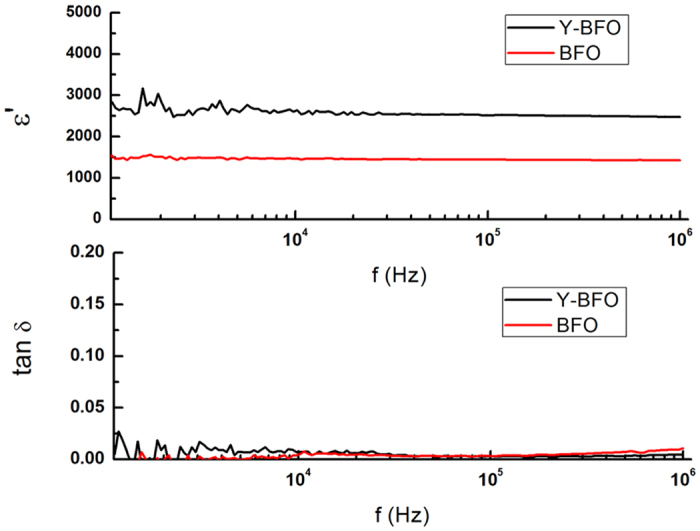
Dielectric constant and loss tangent values measured on Y-BFO and BFO samples for comparing purposes.

Dielectric measurements have been made on a second set of Y-BFO films with IDE electrodes with different dimensions and number of fingers and the results are similar which rule out possible effects of geometric configuration on the measurements (see Supplementary Information). For comparison we have also investigated the dielectric properties of pure BFO films on which similar IDE electrodes have been deposited. Typical results (as obtained on many IDE electrodes) are shown in Fig. 9, as well as in the Supplementary Information. Although the dielectric constant shows a high value, it is however much smaller than for Y-BFO films.

We have analyzed above the effects of strain on the Y-BFO films, which manifest by the appearance of a stripe nanodomain structure with monoclinic symmetry. Stripe-like domain patterns have been identified also in other ferroelectric^37^ or relaxor-ferroelectric systems^38^ and in BFO films grown on different substrates^39^,^40^.

We believe that this peculiar nanostructure could be at the origin of the observed huge dielectric response. However we need to analyze also the influence of small radius Y^3+^ ions on the microstructure and dielectric response of Y-BFO films, by taking advantage of the reported findings on the universal phase diagram of RE-substituted BFO^17^. In Fig. 1(c) the plot lines represent schematically the phase diagram temperature (T)-average ionic radius (R_av_) of Bi_1−x_RE_x_FeO_3_, as proposed in ref. 17. The region enclosed between the continuous lines, separating the main phases ferroelectric *R3c* and paraelectric *O* Pnma, is the bridging mixed phase formed by nanometer-size antipolar domains in the *R3c* matrix^16^,^19^, which is thought to be responsible for the enhancement of the dielectric and electromechanical properties in these compositions. In order to transpose the diagram in terms of compositions, we have estimated the dependence of R_av_ on the dopant concentration x for the different RE ionic radii, including Y^3+^. The corresponding ionic radii of the trivalent A ions in 12-fold coordination as given in refs 17,41,42 are: R_Bi_ (1.36 Å) >R_Sm_ (1.28 Å) >R_Gd_ (1.27 Å) >R_Dy_ (1.24 Å)>R_Y_ (1.22 Å). For every Bi_1−x_RE_x_FeO_3_ composition the average ionic radius is plotted against x in Fig. 1(d). The horizontal lines mark the boundaries of the MPB region (near room temperature) as identified in Fig. 1 (c). The square symbols on the Sm, Gd and Dy lines identify the compositions where the maxima of the dielectric and electromechanical responses have been obtained, as reported in ref. 17. The corresponding value of R_av_ is called critical (R_av,c_) since it is common for all compositions, marking the transition from the R3c to the Pnma phase. Its significance is related to the difference in the free energy density between these two phases, ΔU (R_av_), which is given by the relationship^17^


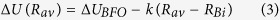


where ΔU_BFO_ is the energy density difference for pure BFO and *k* is a constant. At the critical average ionic radius, R_av_ **=** R_av,c_, ΔU (R_av,c_) **=** 0.

It can be observed that the corresponding compositions are placed within the limits of the MPB region. Moreover, Y-BFO films composition (x **=** 0.05), represented by the square point on the Y line and by the red square symbol in Fig. 1(c), is also inside the MPB region of the universal phase diagram. The smaller the ionic radius of the A-site RE dopant, the lower the concentration needed to induce the new phase. This is easy to understand if one recalls that the *O* Pnma phase is favoured by tilt distortions of the oxygen octahedra which, at their turn, are promoted by small size ions occupying the A-site positions. It has been previously evidenced^16^,^17^,^18^,^19^ that such compositions show also the presence of nanodomains with different orientations which favor the enhancement of dielectric and piezoelectric response, by providing a low energy pathway for polarization rotation between different polar axes. As we have shown above, the presence of nanodomains is characteristic also for Y-BFO epitaxial films. Indeed, strain nanopatterns in geometric phase analysis are related to lattice distortions^32^, which in turn are associated in polar materials with local polarization variations^32^, therefore with nanodomains. One can use basically two approaches to correlate the dielectric permittivity ε to the peculiar nanodomain configuration. The first one is through the averaged polarization response to an electric field E:


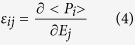


where <P_i_> is the domain-averaged polarization. Although a realistic model is difficult to construct, a simple case of a mixture of two stripe domain variants with different populations α and 1-α has been considered in ref. 43 to obtain the following relation:


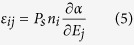


where P_s_ is domain saturation polarization and n_i_ is related to the polarization directions. It is evident from this equation that contributions arising from variations of populations of different nanodomains with electric field will greatly enhance the dielectric response. This has been indeed observed for different polar materials with nanodomain configurations^44^,^45^.

The second approach is through the fluctuation-dissipation theorem^46^, relating the dielectric permittivity to polarization fluctuations. Consider a ferroelectric with two coexisting phases with small energy difference. In this case the fluctuations of polarization occur mainly due to the libration (rotation) of the polarization vector^47^ and they can be expressed as 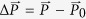, where 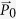 is the easy polarization direction. These rotational fluctuations can be characterized by the thermodynamic average 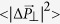, where 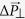 is the projection of 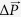 on the plane which is normal to the easy polarization direction. In ref. 47, Khachaturyan has shown that in the first approximation the thermodynamic average of polarization fluctuations can be written as


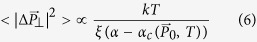


where ξ is a constant, α is the concentration of one of the coexisting phases and α_c_ is the critical concentration signaling the metastability limit, where the energy difference between the coexisting phases is minimum. It means that in the metastability region the domain wall energy γ, which is determined by this energy of polarization change within the domain boundary, becomes minimum and therefore nanodomains are formed, since the following relation


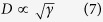


holds between γ and the domain size D. Furthermore, through the fluctuation-dissipation theorem, the dielectric permittivity is related to the averaged polarization fluctuations by the relationship


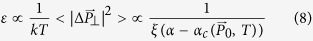


which means that the dielectric permittivity of the material diverges in the metastability limit.

We have seen that, in Y-BFO, the conditions for coexisting phases and nanodomain formations are created by Y-doping and epitaxial strain. We can infer therefore that the high dielectric permittivity of the Bi_0.95_Y_0.05_FeO_3_ thin films is due to the nanoscale complex structure associated with the presence of competing phases at the MPB, promoted by both chemical pressure (Y-doping) and misfit strain caused by the epitaxial growth on the STO substrate.

## Conclusions

We have obtained very high in-plane dielectric permittivity (ε′∼2500) and low dielectric loss (tanδ<0.01) on Y-doped BiFeO_3_ films epitaxially grown on SrTiO_3_(001) by pulsed laser deposition. Our experimental results reveal that the previously evidenced limitations on these values can be surmounted in high quality epitaxial films by promoting the formation of mixed states at nanoscale level. High resolution transmission electron microscopy and geometric phase analysis evidenced nanostripe domains with alternating compressive and tensile strain. Our work brings insight into the combined role of chemical pressure and epitaxial strain on the destabilization of ferroelectric phase and appearance of nanoscale stripe structure.

## Methods

The films were grown by pulsed laser deposition on (001) SrTiO_3_ (STO) single crystal substrates, from ceramic targets with composition Bi_0.95_Y_0.05_FeO_3_ (Y-BFO) and BiFeO_3_ (BFO). The thickness of the films was set around 100 nm, in order to preserve the strained condition. The deposition has been made under 13 Pa oxygen partial pressure, by using an ArF excimer laser (193 nm wavelength) with pulse frequency 5 Hz. The laser pulse energy was 20 mJ and the substrate temperature was 700 ^o^C. In these conditions the growth rate was about 0.1 Å/s.

X-ray diffraction (XRD) at room temperature was performed with a PANalytical X’Pert MRD diffractometer, by using a line focused parallel monochromatic beam with CuKα1 radiation (0.1540598 nm) provided by a hybrid monochromator 2xGe(220) asymmetric.

The HR-TEM investigations have been performed on a JEM ARM 200F microscope.

Piezoforce microscopy (PFM) measurements have been carried out on a commercial AFM (XE-100, Park Systems) in order to evidence the local ferroelectric domain orientation.

For dielectric measurements two sets of Au interdigital electrodes with different dimensions have been deposited on top surfaces of the films by lift-off technique. Low-signal dielectric spectroscopy measurements have been carried out by using an HP4194A impedance bridge equipped with a special holder for contacting the IDE electrodes. In order to verify the correctness of the measurement method and of the calculation model, IDE structures have been deposited also directly on the STO substrate, and its dielectric constant has been measured and compared with datasheet value.

## Additional Information

**How to cite this article**: Scarisoreanu, N. D. *et al*. Joining Chemical Pressure and Epitaxial Strain to Yield Y-doped BiFeO_3_ Thin Films with High Dielectric Response. *Sci. Rep.* **6**, 25535; doi: 10.1038/srep25535 (2016).

## Supplementary Material

Supplementary Information

## Figures and Tables

**Table 1:** Structural data extracted from XRD analysis.

Sample	*c*(Å)	*a*(Å)	*c/a*	Microstrain *ε* ⊥ (%)	RK(200) *ω-*scan (deg)	*L*_||_ (nm)	*α*_tilt (deg)_
Y-BFO	4.0042	3.9263	1.0198	0.322	0.7829	70	0.8615
BFO	3.9983	3.9600	1.0097	0.199	0.5895	239	0.5195
