# Supplementary material for: Joining Chemical Pressure and Epitaxial Strain to Yield Y-doped BiFeO3 Thin Films with High Dielectric Response
Source: Sci Rep. 2016 May 9;6:25535. doi: 10.1038/srep25535 (PMC4860595; doi:10.1038/srep25535)
Supplement: Supplementary Information [file srep25535-s1.pdf]

Supplementary Information

Joining Chemical Pressure and Epitaxial Strain to Yield Y-doped $BiFeO_3$ Thin Films with High Dielectric Response

N. D. Scarisoreanu[1*], F. Craciun[2], R. Birjega[1], V. Ion[1, 4], V. Teodorescu[3], C. Ghica[3], R. Negrea[3], M. Dinescu[1]

[1]*National Institute for Laser, Plasma and Radiation Physics, 077125 Magurele, Romania*

[2]*CNR-ISC, Istituto dei Sistemi Complessi, Area della Ricerca di Roma-Tor Vergata, Via del Fosso del Cavaliere 100, I-00133 Rome, Italy*

[3]*National Institute of Material Physics, 077125, Magurele, Romania*

[4]*Faculty of Physics, University of Bucharest, 077125 Magurele, Romania*

E-mail: nicu.scarisoreanu@inflpr.ro

Contents:

Dielectric constant and loss of used $SrTiO_3$ substrate.

Structural difference details between Y-BFO and pure BFO films.

Dielectric constants and losses for Y-BFO and BFO thin films measured for different interdigital electrodes configurations.

References.

Dielectric constant and loss of the used $SrTiO_3$ substrate

The dielectric constant and loss of the $SrTiO_3$ (STO) substrate have been obtained by depositing interdigital electrodes on their surfaces and measuring the capacitance and loss [1, 2]. Their values have been extracted by using the method described in the main text [3]. The results are shown in the Figure S1. The dielectric constant of STO varies between about 300 and 310 in the frequency range 1 kHz-1 MHz, while tan δ is about 0.005, very similar to the datasheet values given by the producer.

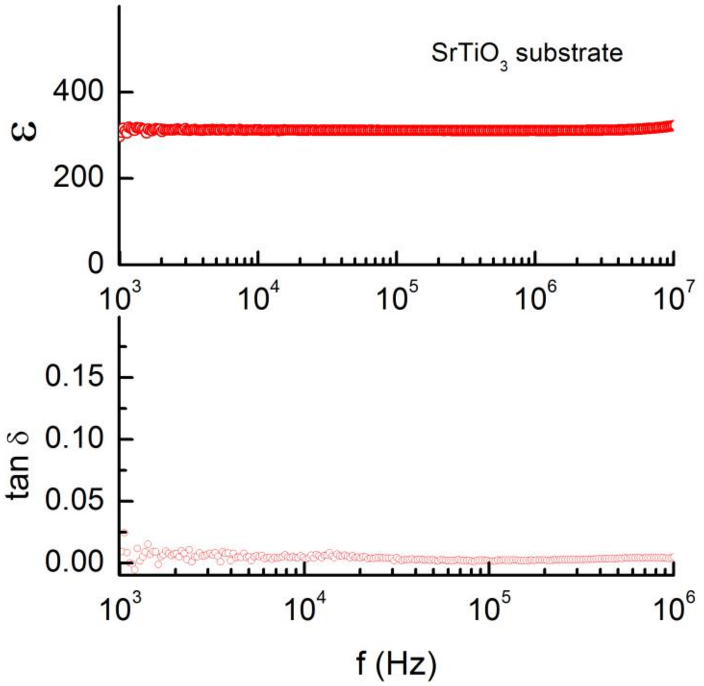

Figure S1. Dielectric constant and loss of STO substrate measured with interdigital electrodes.

Structural difference details between Y-BFO and pure BFO films.

For reproducibility purposes, the structural properties of two sets of Y-BFO thin films have been obtained by XRD measurements and compared with those of pure BFO thin film. The superimposed XRD patterns of the Y-BFO (film 2) in comparison with the Y-BFO (film 1) and the reference BFO-film are presented in Figure S2. The inset image represents the detail of the (004) reflections patterns. The superimposed rocking curves of the (002) peaks are presented in Figure S3. As the two figures reveal, the XRD pattern of the Y-BFO (film 2) almost replicates the XRD pattern

of the Y-BFO (film 1). The structural data are presented in Table S1. Y-BFO (film 2) exhibits the same characteristics in comparison with BFO film: high microstrain value, larger tilt angles and significant decrease of the lateral coherence length.

.

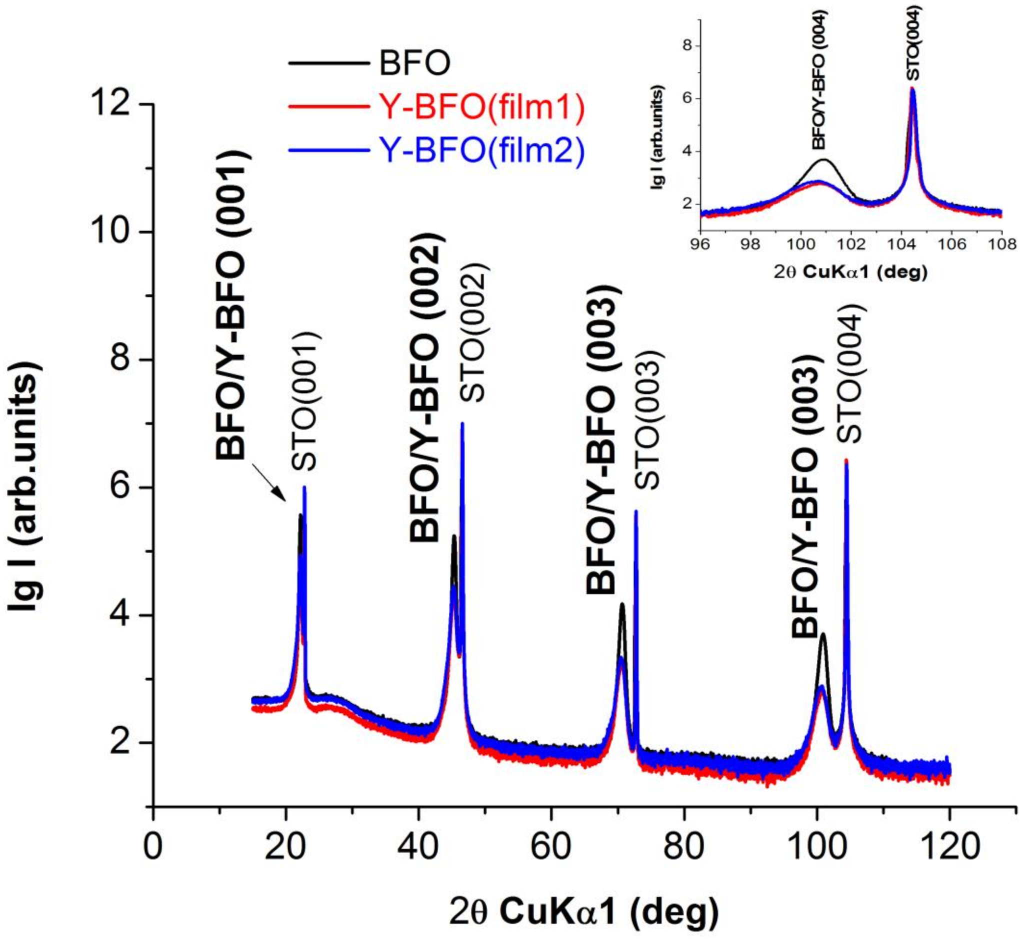

Figure S2. The superimposed XRD patterns of the two Y-BFO films and pure BFO film. Inset: the superimposed (004) reflections patterns.

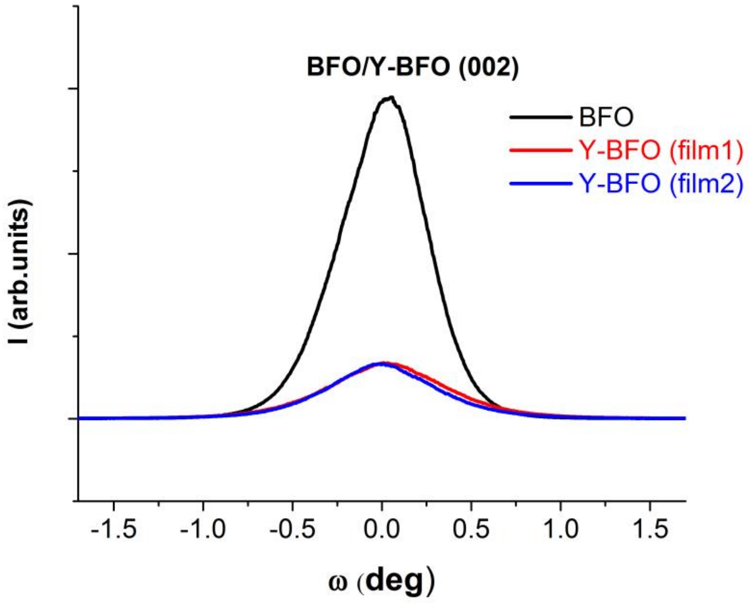

Figure S3. Superimposed rocking curves of the (002) diffraction peaks for two Y-BFO and pure BFO films.

Sample	*c* (Å)	*a* (Å)	*c/a*	Microstrain $\varepsilon\perp$ (%)	RK(200) *w*-scan (deg)	$L_\parallel$ (nm)	α_{tilt} (deg)
Y-BFO (film1)	4.0042	3.9263	1.0198	0.322	0.7829	70	0.8615
Y-BFO (film 2)	4.0062	3.9266	1.0203	0.315	0.7141	42	0.8648
BFO	3.9983	3.9600	1.0097	0.199	0.5895	239	0.5195

Table S1. Structural data extracted from XRD analysis.

Dielectric constants and losses for Y-BFO and BFO thin films measured for different interdigital electrodes configurations

To rule out possible effects of interdigital electrodes (IDE) geometric configuration, dielectric measurements have been performed on a second set of Y-BFO films with IDE electrodes with different dimensions and different number of fingers yielded similar results (Fig. S4). For comparison we have also investigated the dielectric properties of pure BFO films on which similar IDE electrodes have been deposited by the same lift-off technique. The results (as obtained on many

IDE electrodes) are shown in Fig. S4. Although the dielectric constant shows a high value for BFO film, it is much smaller than for Y-BFO films in any IDE geometric configuration.

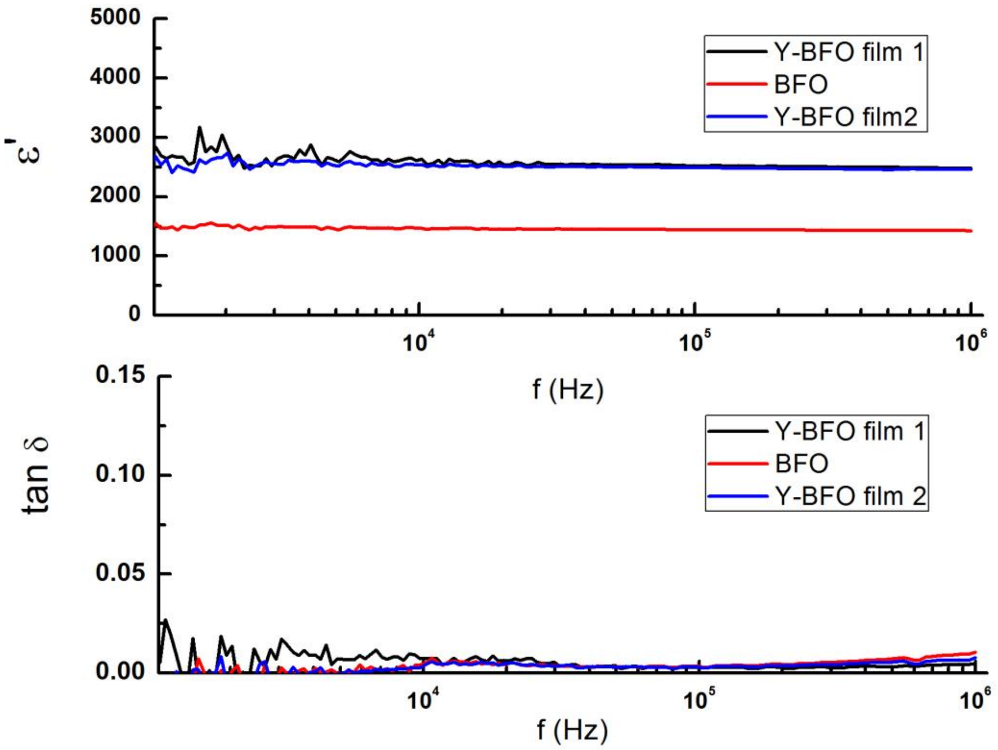

Figure S4. Dielectric constant and loss tangent values measured on two sets of Y-BFO films with IDE electrodes having different dimensions and number of fingers; the values for the Y-BFO films are presented together with those obtained for pure BFO films.

References

1. G. W. Farnell, I. A. Cermak, P. Silvester, S. K. Wong, *IEEE Trans. on Sonics and Ultrasonics SU-17*, **1970**, 188.
2. H. N. Al-Shareef, D. Dimos, M. V. Raymond, R. W. Schwartz, C. H. Mueller, *J. Electroceram*. **1997**, 1:2, 145.
3. N. D. Scarisoreanu, F. Craciun, A. Moldovan, V. Ion, R. Birjega, C. Ghica, R. F. Negrea, M. Dinescu, *ACS Appl. Mater. Interfaces,* **2015**, 7, 23984−23992.
